# Nationwide molecular epidemiology of carbapenemase-producing *Citrobacter* spp. in France in 2019 and 2020

**DOI:** 10.1128/msphere.00366-23

**Published:** 2023-10-10

**Authors:** Laura Biez, Rémy A. Bonnin, Cecile Emeraud, Aurélien Birer, Agnès B. Jousset, Thierry Naas, Laurent Dortet

**Affiliations:** 1 Team "Resist" UMR1184 "Immunology of Viral, Auto-Immune, Hematological and Bacterial diseases (IMVA-HB)," INSERM, Université Paris-Saclay, CEA, LabEx LERMIT, Faculty of Medicine, Le Kremlin-Bicêtre, France; 2 Associated French National Reference Center for Antibiotic Resistance: Carbapenemase-Producing Enterobacteriaceae, Le Kremlin-Bicêtre, France; 3 Bacteriology-Hygiene Unit, Assistance Publique-Hôpitaux de Paris, AP-HP Paris Saclay, Bicêtre Hospital, Le Kremlin-Bicêtre, France; 4 Centre National de Référence de la Résistance aux Antibiotiques, Clermont-Ferrand, France; University of Nebraska Medical Center, Omaha, Nebraska, USA

**Keywords:** Citrobacter, carbapenem, antibiotic, resistance, MLST, core genome

## Abstract

Carbapenemase-producing Enterobacterales have emerged in the last decades as one of the main threats in modern medicine. *Enterobacter* is a genus increasingly recognized as a key player in carbapenemase diffusion besides *Escherichia coli* and *Klebsiella pneumoniae*, but information regarding *Citrobacter* spp. remained scarce. A collection of 803 isolates of *Citrobacter* spp. recovered from the French National Center for carbapenem resistance over a 2-year period during 2019–2020 was analyzed. A total of 15 different carbapenemases were identified of which OXA-48 followed by NDM-1 and OXA-181 represented the main enzymes. Phylogenetic analysis of this collection revealed that *Citrobacter freundii* was the main species among which three main clones are circulating in France, being ST8, ST22, and ST91. ST22 and ST8 were distributed in all regions whereas ST91 demonstrated a regional spread. Analysis of the species diversity revealed that among the *C. freundii* complex, *Citrobacter portucalensis* and *Citrobacter braakii* were also disseminated. We revealed the dissemination of a clone carrying both *bla*_DHA-15_ (β-lactamase-encoding gene rarely reported in Enterobacterales) and *bla*_OXA-48_ in the north of France and allowed to identify an outbreak not previously investigated. This study analyzed the largest collection of carbapenemase-producing *Citrobacter* spp. and allowed deciphering of molecular epidemiology of *Citrobacter* spp. in France.

**IMPORTANCE:**

The emergence of carbapenemase producers in Enterobacterales mostly involves *Escherichia coli*, *Klebsiella pneumoniae*, and *Enterobacter cloacae* complex species. However, in France, we observed the emergence and the rapid dissemination of carbapenemase in *Citrobacter* spp. In this study, we demonstrated that a wide variety of carbapenemases is produced by many different species of *Citrobacter* spp. However, we clearly identify three high-risk clones of *Citrobacter freundii*, ST8, ST22, and ST91 that drive the spread of carbapenemase in France. This epidemiological study paves the way of further analysis that would aim to identify the virulence factors involved in this pellicular ability of these three clones to disseminate at the hospital.

## INTRODUCTION

*Citrobacter* spp. are facultative anaerobic Gram-negative rod-shaped bacteria belonging to Enterobacterales initially named according to their utilization of citrate as carbon source (1, 2). *Citrobacter* species are found in soil, water, and food and are commensal of the digestive tract (3). They are opportunistic pathogens able to induce urinary tract infections, neonatal sepsis, meningitis inducing brain abscess, respiratory tract infections, gastrointestinal infections, and central nervous system infections, in neonates and in immunocompromised patients (4).

*Citrobacter freundii* was initially named *Bacterium freundii* in 1928. In 1932, it was reclassified into the genus *Citrobacter* that contains now 16 named subspecies: *C. freundii*, *Citrobacter braakii*, *Citrobacter gillenii*, *Citrobacter murliniae*, *Citrobacter rodentium*, *Citrobacter sedlakii*, *Citrobacter werkmanii*, *Citrobacter youngae*, *Citrobacter koseri* (formerly known *Citrobacter diversus*), *Citrobacter farmeri*, *Citrobacter amalonaticus*, *Citrobacter pasteurii*, *Citrobacter europaeus*, *Citrobacter portucalensis*, *Citrobacter cronae*, and *Citrobacter telavivensis* (https://lpsn.dsmz.de/genus/citrobacter).

Inside this genus*, C. koseri* is genetically distant from *C. freundii* and the other subspecies (5). Moreover, *C. koseri* belongs to the group II Enterobacterales, as it produces a class A chromosomally encoded penicillinase that confers resistance to amino-, carboxy-, and ureido-penicillins (amoxicillin) and is inhibited by clavulanate or tazobactam (6). *C. freundii* and the other *Citrobacter* spp. belong to the group III Enterobacterales as they possess an Ambler class C chromosomally encoded cephalosporinase (*bla*_CMY-like_) conferring an intrinsic resistance to aminopenicillins, to classical β-lactamase inhibitors such as clavulanate or tazobactam, and first-generation cephalosporins (7). Overexpression of *bla*_CMY-like_ genes may lead to resistance to expanded-spectrum cephalosporins and even carbapenems, if associated with decreased permeability (7).

Carbapenem-hydrolyzing class D β-lactamase (CHDL) OXA-48 and to a lesser extent the class B metallo-β-lactamase NDM-1 are the most frequently carbapenemase encountered in carbapenemase-producing Enterobacterales (CPE) isolated in France (8, 9).

The aim of this study was to characterize the epidemiology of carbapenemase-producing *Citrobacter* spp. received at the French National Reference Center (F-NRC) for CPEs in 2019–2020.

## MATERIALS AND METHODS

### Strain collection

A total of 1,121 carbapenem non-susceptible *Citrobacter* spp. were received at the F-NRC for CPEs between January 1st, 2019, and December 31st, 2020. Clinical isolates were firstly identified by MALDI-TOF mass spectrometry (MALDI Biotyper, Wissembourg, France).

### Antimicrobial susceptibility testing and carbapenemase detection

Antimicrobial susceptibility testing was performed using the disc diffusion method on Mueller-Hinton (MH) agar (Bio-Rad, Marnes-La-Coquette, France), and interpreted according to EUCAST guidelines (https://eucast.org/clinical_breakpoints/).

The carbapenemase activity was assessed using the updated Carba NP test as described (10), and the type of carbapenemase was identified by the lateral flow immunoassay (LFIA) NG-Test Carba5 (NG Biotech, Guipry, France) (11).

### Whole-genome sequencing and bioinformatics

Whole-genome sequencing was performed using Illumina’s NextSeq 500 as previously described (12). *De novo* assembly was performed using CLC Genomics Workbench v12.0 (Qiagen, Les Ulis, France). Acquired resistance gene and multilocus sequence typing (MLST) were performed using resfinder 4.1 and MLST 2.0 tools on the Center for Genomic Epidemiology server (http://www.genomicepidemiology.org/). Single nucleotide polymorphism (SNP) analysis and phylogeny were performed as previously described (13).

## RESULTS

### Whole-genome phylogeny

**Fig 1:**
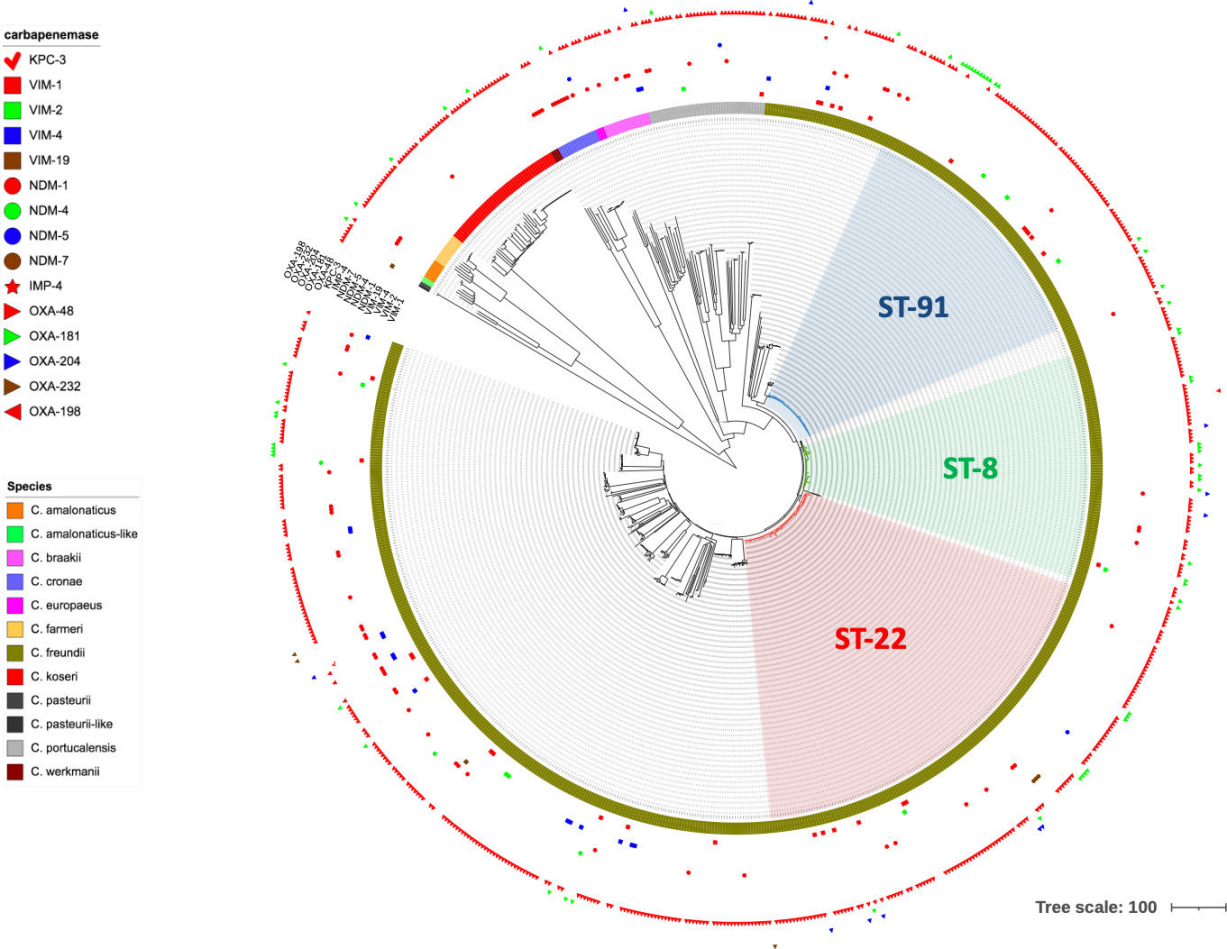
Global phylogeny of carbapenemase-producing *Citrobacter* spp. isolated in France in 2019 and 2020.

To assess the genetic diversity of *Citrobacter* spp., a phylogenetic tree was constructed based on the whole genome of the 803 carbapenemase-producing isolates (Fig. 1). As expected, it revealed that the main species corresponded to *C. freundii sensu stricto*. The main species, apart from *C. freundii*, correspond to *C. koseri* (*n* = 46) followed by *C. portucalensis* (*n* = 42), *C. braakii* (*n* = 17), and *C. cronae* (*n* = 15) (Fig. 1).

However, WGS results revealed that several isolates sent to the F-NRC were incorrectly identified as *C. freundii* using MALDI-TOF, including isolates of *C. europaeus*, *C. portucalensis*, *C. cronae*, and *C. werkmanii* (Fig. 1) This is likely due the inability of MALDI-TOF to distinguish between these *C. freundii* complex species. The more distant species, and correctly identified by MALDI-TOF, included *C. koseri*, *C. farmeri*, *C. amalonaticus*, and *C. pasteurii*. Among the two *C. pasteurii* identified, one was very distant and could be considered as *C. pasteurii*-like. Further characterizations are needed to decipher if it truly belongs to *C. pasteurii* or to a novel species.

### MLST analysis

MLST analysis evidenced three predominant clones, being ST8, ST22, and ST91, all from *C. freundii stricto senso* (Fig. 1). These sequence types (STs) represent 40.0% of total isolates with 78, 145, and 98 isolates belonging to ST8, ST22, and ST91, respectively (Fig. 1).

**Fig 2:**
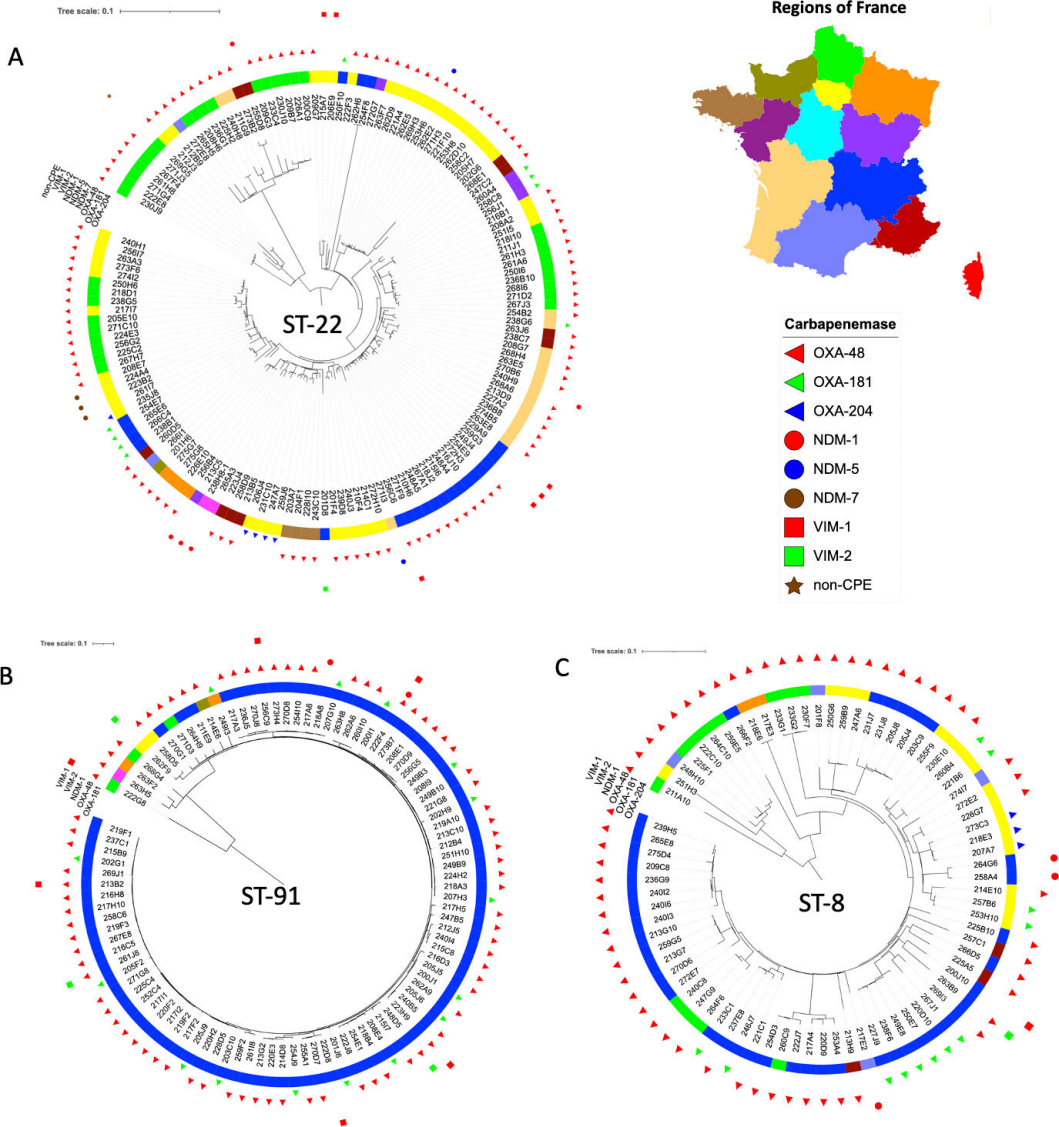
Phylogeny of the three main clones of *C. freundii*, ST22 (panel A), ST8 (panel B), and ST91 (panel C).

*C. freundii* isolates of ST22 were the most prevalent (Fig. 1). They were mostly recovered from three regions being Paris area (Ile-de-France, *n* = 45), north-east of France (Hauts-de-France, *n* = 22), and south-east (Auvergne-Rhone-Alpes, *n* = 22). However, isolates belonging to ST22 were also from French island such as Réunion Island (Indian Ocean) (Fig. 2). The in-depth analysis of the phylogenetic tree of ST22 revealed a wide genetic diversity inside this ST. Indeed, the size of the branches demonstrated a broad genetic diversity (Fig. 2A). As an example, despite being part of the ST22, some isolates seemed more distantly related (e.g., 262H6 and 254F8 isolates). This genetic diversity might also be highlighted by the presence in the same branch of clearly unrelated isolates recovered from different regions. Inside this genetic diversity, few outbreaks can be evidenced, such as 9 OXA-48-producing isolates recovered from an outbreak in Hauts-De-France or 14 clonally related strains in the region of Lyon (highlighted in red in Fig. 2A). Regarding this last outbreak which involved 14 isolates in Lyon, it is worthy to note that 3 isolates produced VIM-1, 10 isolates produced OXA-48, and the remaining isolate co-produced both carbapenemases. It suggests a wide spread of this particular clone that independently or successively acquired VIM-1 and OXA-48 carbapenemases in a restricted area (same hospital).

The second most prevalent cluster corresponds to ST91 (98 isolates). Of note, 90.8% (89/98) of the ST91 isolates were recovered from the same region (Provence-Alpes-Côte d’Azur) strongly indicating a wide dissemination in this particular area (Fig. 2B). Noticeably, although the *bla*_OXA-48_ gene is the main carbapenemase gene carried by this clone, 15 *bla*_OXA-181_-carrying isolates were also identified. Two isolates carried the carbapenemase-encoding *bla*_VIM-2_ gene. In these two strains, *bla*_VIM-2_ was carried on a plasmid previously identified in *Pseudomonas putida* (GenBank accession number MK047608). In ST91, one isolate produced three carbapenemases being OXA-48, NDM-1, and VIM-1 (14).

The remaining main clone, ST8, has been identified in different regions of France and associated with different carbapenemase (Fig. 2C). It might indicate a wide spread of this clone rather than a recent dissemination as observed for ST91.

### Antimicrobial susceptibility and carbapenemase production

Among the 1,121 carbapenem non-susceptible *Citrobacter* spp. received at the F-NRC for CPEs over the study period, 1,023 isolates produced a carbapenemase as revealed by Carba NP. The OXA-48-like carbapenemases were the most frequent, followed by NDM, VIM, KPC, and IMP as revealed by LFIA and further confirmed by WGS (Table 1).

**TABLE 1:** Distribution of carbapenemases by *Citrobacter* species

Species	*n*	Carbapenemases (*n* = 791)														
		VIM-1	VIM-2	VIM-4	VIM-19	NDM-1	NDM-4	NDM-5	NDM-7	IMP-4	KPC-3	OXA-48	OXA-181	OXA-204	OXA-232	OXA-198
*C. freundii*	643	28	8	16	1	48	3	1	3	1	1	493	56	10	3	1
*C. amalonaticus*	9					3						5	1			
*C. braakii*	17			2		4						11				
*C. cronae*	15					9		1				5	1			
*C. europaeus*	3					1						2				
*C. farmeri*	11											10	1			
*C. koseri*	46					1						42	3			
*C. pasteurii-*like	2				1							2				
*C. portucalensis*	42	1	1			4		1				35	1	1		
*C. werkmanii*	3					2						1				
Total	791	29	9	18	2	72	3	3	3	1	1	606	63	11	3	1

Susceptibility testing was performed on the 1,121 carbapenem non-susceptible *Citrobacter* spp. isolates. All isolates were resistant to ticarcillin and ticarcillin/clavulanate combination, 99.4% were resistant to piperacillin-tazobactam combination, and 97.9% were resistant to temocillin. Resistance rates to cefotaxime, aztreonam, and cefepime were of 86.8%, 72.2%, and 64.7%, respectively. Among these isolates, 91.2% were resistant to ertapenem whereas 11.4% and 13.2% were resistant to meropenem and imipenem, respectively (Table 2). Regarding non-β-lactams, resistance rates to aminoglycosides were of 21.4% for amikacin and 69.6% for gentamicin. Resistance rates to fluoroquinolones were of 81.0% and 73.5% for ciprofloxacin and levofloxacin, respectively (Table 2).

**TABLE 2:** Resistance profiles of *Citrobacter* spp. included in this study

Molecules	Resistance rates (%)		
**β-Lactams**	**R^*a*^**	**I^*a*^**	**S^*a*^**
Ticarcillin	100	N/A^*b*^	0.0
Ticarcillin-clavulanate	100	N/A	0.0
Piperacillin-tazobactam	99.4	N/A	0.6
Temocillin	97.9	2.1	0.0
Mecillinam	32.1	N/A	67.9
Cefotaxime	86.8	2.6	10.6
Ceftazidime	85.2	1.8	13.0
Ceftazidime-avibactam	17.4	N/A	82.6
Ertapenem	91.2	N/A	8.8
Meropenem	11.4	22.0	66.6
Imipenem	13.2	14.8	72.0
**Aminoglycosides**			
Amikacin	21.4	N/A	78.6
Gentamicin	69.6	N/A	30.4
**Fluoroquinolones**			
Ciprofloxacin	81.0	4.8	14.2
Levofloxacin	73.5	5.2	21.3
**Others**			
Tigecycline	7.6	N/A	92.4
Sulfamethoxazole/trimethoprim	65.8	1.1	33.1
Fosfomycin	9.3	N/A	90.7

^*a*^ S, susceptible; I, susceptible at high exposure; R, resistant.

^*b*^ N/A, not available.

Fosfomycin, tigecycline, and ceftazidime/avibactam remained in the susceptibility range for 90.7%, 92.4%, and 82.6%, respectively. Diameter distribution of ceftazidime/avibactam revealed a bimodal distribution with a highly resistant population likely indicating a production of metal-β-lactamase.

### Resistome analysis

Of the 1,023 isolates that produced a carbapenemase, 803 non-duplicate isolates were sequenced. Analysis of their resistomes revealed that the main carbapenemase gene found in these isolates corresponded to *bla*_OXA-48_ (*n* = 606), followed by *bla*_NDM-1_ (*n* = 72) and *bla*_OXA-181_ (*n* = 63) (Table 1). Fifteen different carbapenemase genes were identified during this study including four *bla*_VIM_-like variants [*bla*_VIM-1_ (*n* = 29), *bla*_VIM-2_ (*n* = 9), *bla*_VIM-4_ (*n* = 18), and *bla*_VIM-19_ (*n* = 2)], four *bla*_NDM_-like variants [*bla*_NDM-1_ (*n* = 72), *bla*_NDM-4_ (*n* = 3), *bla*_NDM-5_ (*n* = 3), and *bla*_NDM-7_ (*n* = 3)], *bla*_IMP-4_ (*n* = 1), *bla*_KPC-3_ (*n* = 1), four *bla*_OXA-48_-like variants [*bla*_OXA-48_ (*n* = 606), *bla*_OXA-181_ (*n* = 63), *bla*_OXA-204_ (*n* = 11), and *bla*_OXA-232_ (*n* = 3)], and *bla*_OXA-198_ (*n* = 1).

Besides carbapenemase genes, numerous broad-spectrum β-lactamase-encoding genes were identified including ESBL-encoding genes such as eight different *bla*_CTX-M_ alleles (*bla*_CTX-M-1_, *bla*_CTX-M-3_, *bla*_CTX-M-9_, *bla*_CTX-M-14_, *bla*_CTX-M-15_, *bla*_CTX-M-32_, *bla*_CTX-M-55_, and *bla*_CTX-M-232_), *bla*_GES-1_, *bla*_GES-7_, *bla*_VEB-1_, *bla*_DHA-1_-like, or plasmid-encoded cephalosporinase genes such as *bla*_DHA-1_ or *bla*_DHA-15_.

By contrast with *bla*_DHA-1_ gene identified in multiple STs, *bla*_DHA-15_ gene have only been identified in ST500. Epidemiological data revealed that all these ST500 *C. freundii* isolates were from the same city and thus revealed a loco-regional spread of a single clone. As described in *Morganella*, the natural progenitor of *bla*_DHA_-like genes, *bla*_DHA-15_ is associated to its transcriptional regulator LysR. Analysis of the genetic of the *bla*_DHA-15_ environment revealed the presence of a quinolone resistance protein *qnrB4* close to this gene.

Among ESBLs identified in this collection, *bla*_GES-1_ was present in 10 isolates including 8 *C*. *freundii*, 1 *C. koseri*, and 1 *C. amalonaticus*. This gene was identified in four different STs in the *C. freundii* complex being ST116 (*n* = 4), ST216 (*n* = 2), ST22 (*n* = 1), and ST118 (*n* = 1). Noticeably, the extended-spectrum class D *bla*_OXA-35-like_ was identified in 22 isolates including 20 *C*. *freundii* and 2 *C*. *portucalensis*. Analysis of the genetic background identified a wide genetic diversity of STs (*n* = 10) carrying this gene.

Aminoglycoside resistance genes conferring resistance to gentamicin and amikacin were found in 69.6% and 21.4%, respectively, of the isolates that were resistant to these molecules. Among isolates resistant to both molecules, the 16S RNA methyltransferase-encoding gene *armA* was identified in 19 isolates (14 *C*. *freundii*, 2 *C*. *portucalensis*, 2 *C*. *cronae*, and 1 *C. europaeus*). Two other 16S RNA methyltransferase-encoding genes were identified, *rmtB1* and *rmtC* in 1 *C. freundii* and 16 isolates, respectively (11 *C*. *freundii*, 2 *C*. *braakii*, 2 *C*. *cronae*, and 1 *C. werkmanii*). Noticeably, two isolates harbored a variant of the *tet(X5*) gene. These two isolates correspond to a *C. portucalensis* ST421 and to a *C. freundii* ST22.

Plasmid-encoded colistin resistance genes *mcr9.1* (*n* = 13 isolates) and *mcr9.2* (*n* = 9 isolates) were also identified in different *Citrobacte*r species, suggesting dissemination of these resistance alleles across this genus.

### Outbreak of DHA-15 and OXA-48-producing isolates in northern France

During the analysis of resistomes, some β-lactamase-encoding genes rarely reported in Enterobacterales, but commonly identified in *Pseudomonas* spp., caught our interest such as *bla*_VIM-2_, *bla*_GES-1_, and a rare plasmid-encoded cephalosporinase *bla*_DHA-15_ gene. The *bla*_DHA-15_ gene is present in GenBank database in only two sequences corresponding to the initial description (GenBank accession number NG_049060) and a plasmid-encoded sequence identified in *C. freundii* (GenBank accession number OW969688). In our collection, DHA-15 was present in nine isolates of *C. freundii* belonging to ST500. This ST500 was reported only once in PubMLST database (https://pubmlst.org/) in Poland in 2017.

**Fig 3:**
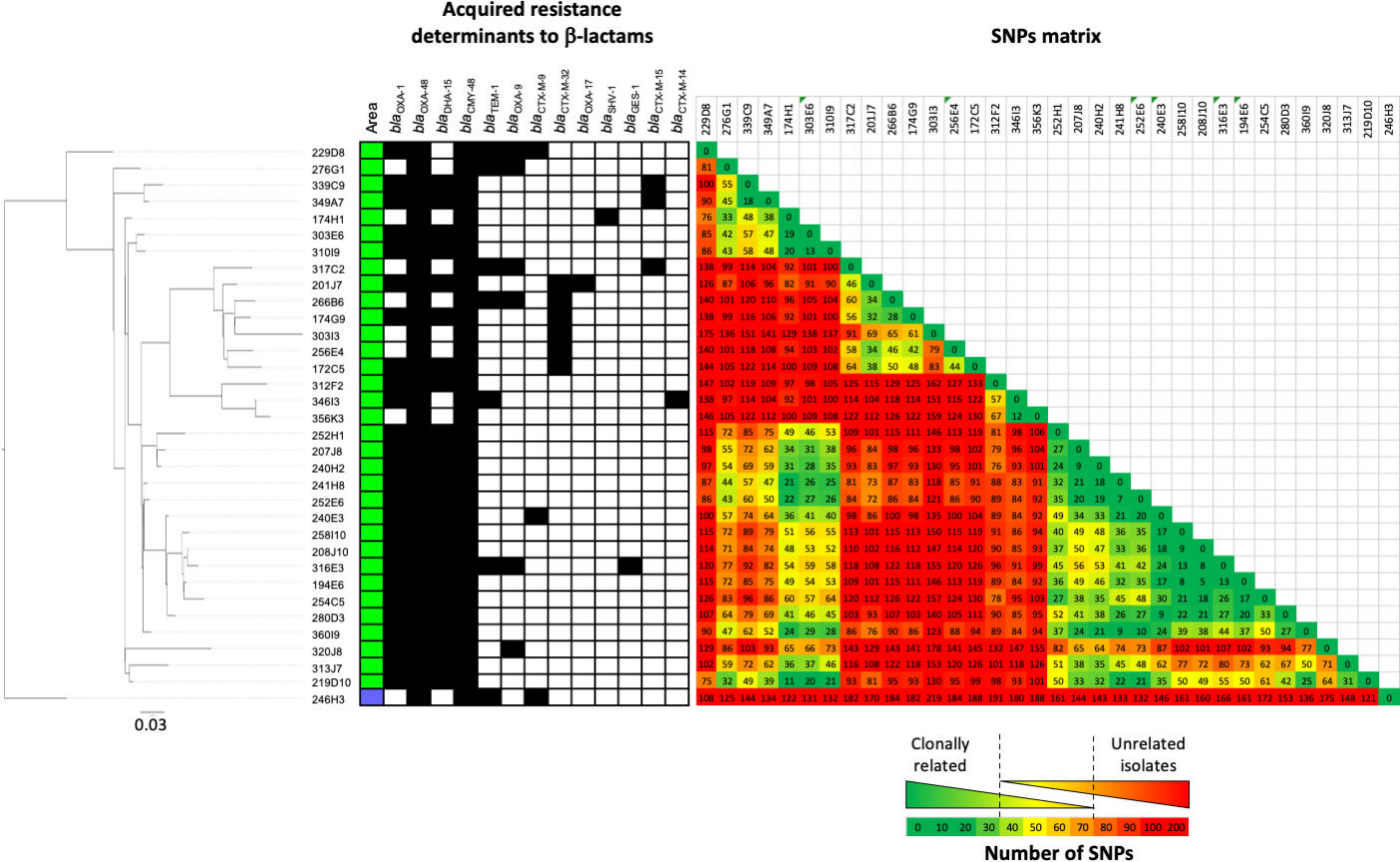
Genomic and phylogenetic characterization of ST500 *Citrobacter freundii*.

To decipher if these DHA-15-producing ST500 *C. freundii* corresponded to a clonal diffusion, we collected all DHA-15-producing isolates and all *Citrobacter* spp. belonging to ST500 from the F-NRC over a 6-year period (2014–2020). A total of 34 *C*. *freundii* of ST500 were identified. Among them, 97.1% (33/34) were isolated in the same region in the north of France (Fig. 3). The phylogenetic analysis identified a polyclonal dissemination of several clones producing all the OXA-48 carbapenemase associated with a wide diversity of other β-lactamases (24 DHA-15-, 6 CTX-M-32-, 1 SHV-1-, 1 GES-1-, 3 CTX-M-9-, 3 CTX-M-15-, and 1 CTX-M-14-producing isolates, respectively).

## DISCUSSION

This study characterized the epidemiology of carbapenemase-producing *Citrobacter* spp. at a genomic level and identified the most prevalent clones that circulated in France in 2019 and 2020. A total of 803 phenotypically characterized isolates were whole-genome sequenced.

Susceptibility testing revealed that among carbapenemase-producing isolates, 8.8%, 66.6%, and 72.0% remained susceptible to ertapenem, meropenem, and imipenem, respectively. Ceftazidime/avibactam combination remained an effective combination except for metallo-β-lactamase producers for which high-level resistance was observed as previously described (15).

Analysis of the resistome revealed that besides the wide dissemination of the *bla*_OXA-48_ gene and into a lesser extent *bla*_NDM-1_, an unexpected high diversity of other beta-lactamases was identified in this collection. This includes the production of VIM-2 rarely described in Enterobacterales (16) and commonly identified in *Pseudomonas* spp. (17). Surprisingly, analysis of the genetic context surrounding the *bla*_VIM-2_ gene revealed a close genetic context with a plasmid identified in *P. aeruginosa* suggesting a genetic transfer between these two genus. Another particularity is the presence of the *bla*_GES-1_ gene in 10 isolates belonging to different *Citrobacter* species and the dissemination of the *bla*_DHA-15_ gene that seems to be correlated to the spread of *C. freundii* ST500 and responsible of several outbreaks in the north of France.

Analysis of the clones circulating in France revealed that three main clones were disseminating carbapenemase being ST8, ST22, and ST91. Despite that these clones are more prevalent, the drivers for their dissemination differ. Indeed, ST91 dissemination corresponded to a clonal expansion mainly within a single region whereas ST8 and ST22 seem to have disseminated in the entire country with a more genetic diversity. In addition, the dissemination of these two STs (ST8 and ST22) is uncorrelated to the production of a given carbapenemase suggesting that these STs can be defined as “high-risk” clones of multidrug resistance. This is the main caveat of this study. We should acknowledge that our collection included only carbapenemase-producing *Citrobacter* spp. isolates. To really define ST8 and ST22 as a high-risk clone, it will be needed to study antibiotic-susceptible *Citrobacter* spp. or ESBL producers, too. This additional investigation might help differentiate clones involved in carbapenemase dissemination from those that are intrinsically highly prevalent. Our results pave the way of the analysis of core genomes of these highly prevalent STs to identify features that might be associated with their success.

Finally, as previously observed for other Enterobacterales such as the *Enterobacter cloacae* complex (18–20), we demonstrated that MALDI-TOF is not precise enough to accurately identify *C. freundii* complex isolates at the species level. It might be of interest to use our collection of deeply characterized *Citrobacter* spp. isolates to increase the MALDI-TOF database to see whether specific markers may help separate these species.

## Data Availability

All genome sequences are stored on the F_NRC webserver. These sequences are available on demand from the corresponding author.
